# A web platform for landuse, climate, demography, hydrology and beach erosion in the Black Sea catchment

**DOI:** 10.1038/sdata.2017.87

**Published:** 2017-07-04

**Authors:** Anthony Lehmann, Yaniss Guigoz, Nicolas Ray, Emanuele Mancosu, Karim C. Abbaspour, Elham Rouholahnejad Freund, Karin Allenbach, Andrea De Bono, Marc Fasel, Ana Gago-Silva, Roger Bär, Pierre Lacroix, Gregory Giuliani

**Affiliations:** 1 University of Geneva, Institute for Environmental Sciences, enviroSPACE Lab., Bd. Carl-Vogt 66, Geneva CH-1211, Switzerland; 2 University of Geneva, Department F.-A. Forel of Environmental and Aquatic Sciences, Bd. Carl-Vogt 66, Geneva CH-1211, Switzerland; 3 University of Geneva, Institute for Environmental Sciences, GRID-Geneva, Bd. Carl-Vogt 66, Geneva CH-1211, Switzerland; 4 European Topic Centre-Spatial Information and Analysis, University of Malaga, edificio CAITI, Campus de Teatinos, Malaga 29071, Spain; 5 Swiss Federal Institute of Aquatic Science and Technology, Ueberlandstrasse 133, Duebendorf CH-8600, Switzerland; 6 Department of Environmental Systems Science, ETH Zurich, Universitaetstrasse 16, Zurich CH-8092, Switzerland; 7 Laboratory of Hydrology and Water Management, Ghent University, Ghent B-9000, Belgium; 8 University of Bern, Centre for Development and Environment, Hallerstrasse 10, CH-Bern 3012, Switzerland

**Keywords:** Hydrology, Environmental sciences, Climate sciences, Geography

## Abstract

The Black Sea catchment (BSC) is facing important demographic, climatic and landuse changes that may increase pollution, vulnerability and scarcity of water resources, as well as beach erosion through sea level rise. Limited access to reliable time-series monitoring data from environmental, statistical, and socio-economical sources is a major barrier to policy development and decision-making. To address these issues, a web-based platform was developed to enable discovery and access to key environmental information for the region. This platform covers: landuse, climate, and demographic scenarios; hydrology and related water vulnerability and scarcity; as well as beach erosion. Each data set has been obtained with state-of-the-art modelling tools from available monitoring data using appropriate validation methods. These analyses were conducted using global and regional data sets. The data sets are intended for national to regional assessments, for instance for prioritizing environmental protection projects and investments. Together they form a unique set of information, which lay out future plausible change scenarios for the BSC, both for scientific and policy purposes.

## Background & Summary

The data sets presented in this paper were essentially generated in the framework of a European research project entitled ‘EnviroGRIDS: Building Capacity for a Black Sea Catchment Observation and Assessment System supporting Sustainable Development’ that took place between 2009 and 2013 with the participation of more than one hundred scientists from thirty different partner institutions from fifteen countries^1^.

This project addressed the gap between scientific information available in the Black Sea catchment (BSC) and the environmental policy needs of the Commission for the Protection of the Black Sea against Pollution and the International Commission for the Protection of the Danube River^2^. The main aim of the project was to build capacity on new Earth Observation techniques and data sharing through web services as a European contribution to the Global Earth Observation System of Systems (GEOSS)^3^. In order to demonstrate the benefit of system interoperability and data sharing, the project developed a pipeline of analyses that produced many new and original data sets for the Black Sea (400,000 km^2^) and its entire hydrological catchment (2.2 mio. km^2^).

**Figure 1:**
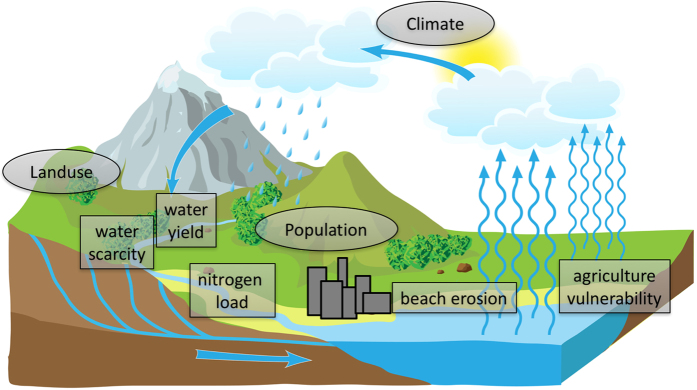
EnviroGRIDS suite of analyses: plausible landuse, climatic and demographic scenarios (circles); SWAT hydrological modelling; water scarcity and vulnerability analyses; and beach erosion (squares).

The enviroGRIDS project is rooted in and took its name from the intended use of computer grid technology to process large amount of environmental data through complex models^4^. This data processing followed a logical suit of analyses that is illustrated in Fig. 1.

The first part of the analyses aimed at setting the scene in terms of plausible climatic, demographic and landuse change scenarios for the entire BSC until 2050. Regionalized storylines were first developed to define four scenarios (BS-Hot, BS-Alone, BS-Coop, BS-Cool) according to their position on two axes of globalisation-regionalisation on the one hand, and liberalisation-policy control on the other hand. Climatic scenarios were derived from the Delta method in order to extrapolate temperature and precipitation according to different scenarios from the Intergovernmental Panel on Climate Changes (IPCC). Demographic scenarios were developed on the basis of the extrapolation census data by political units with urban and rural models^5^. Finally, landuse change was modelled with the Metronamica^6^ software with an iterative yearly stochastic process taking into account the probability of change from one landuse to another and the accessibility by roads and trains^7^.

The second set of analyses is linked to hydrological models using the Soil and Water Assessment Tools (SWAT2009; ref. 8). This model was used to model and predict river flow and nutrient loads (NO_3_) into the Black Sea.

A third independent analysis was done on the outputs from the SWAT model that were used to assess agricultural water vulnerability^9^ and blue water scarcity^10^.

Finally, beach erosion was modelled along the entire Black Sea coastline and across six countries by first digitizing manually every beach based on background satellite images, and then applying an ensemble of six erosion models on them^11^.

This coherent set of environment geospatial data sets on the BSC constitutes a regional contribution of the enviroGRIDS project to the GEOSS. These data sets and their corresponding metadata are made freely available (1) through direct download, (2) as web services, and (3) as map composition to ease visualisation on a GeoNode platform (http://blacksea.grid.unep.ch) that is a simple implementation of a typical Spatial Data Infrastructure^4^. Full copies of these data sets and metadata have been uploaded to Dryad (Data Citation 1).

## Methods

The setting of spatially explicit **landuse change** scenarios has been defined from qualitative socio-economical enviroGRIDS storylines^7^ (BS-HOT, BS-ALONE, BS-COOP and BS-COOL) based on the interpretation of the respective global scenarios from the IPCC (A1, A2, B1 and B2). Quantitative statistical downscaling techniques were used to disaggregate the outputs from global scenarios (IMAGE^12^, version 2.2) at the level of administrative units (Nomenclature of Units for Territorial Statistics, level 2—NUTS2). The disaggregation of the landuse statistics has been divided into three economic groups: FSU (Former Soviet Union), OECD WEU (OECD members in Western Europe including Turkey) and REF EEU (economic reform countries in Eastern Europe). The resulting landuse indicators were spatially distributed at a 1 km resolution by an allocation model (Metronamica^6^) applying a set of factors related to the identified driving forces of change. The landuse change model^13^ was calibrated by integrating spatial allocation rules translated from historical trends of landuse change (MODIS 2001 and 2008), by considering a defined stochastic perturbation, by applying suitability and constraint maps, and by respecting population trends downscaled from National statistical offices to regional (NUTS2) level^5^ (see demographic section below). Physical suitability describes how suited a cell is to a specific landuse type, based on the overlay of different variables such as elevation, slope, soil quality^14^, mean temperature and annual precipitation (from WorldClim^15^). The constraint maps were used to limit or stimulate particular changes or permanence in landuse by assigning a potential change level to each individual class. In detail, protected areas (Natura 2000, EEA; WDPA, UNEP/GRID-Europe), fire events and flood risk, based on the 2009 Global Assessment Report on Disaster Risk Reduction^16^, were used to constrain the urban sprawl. Conversely, major and secondary roads from the Global Road Inventory Project and ESRI, as well as rail network, were included as accessibility maps to favour urban sprawl. The total potential derived by the combination of the different factors was calibrated measuring the goodness of fit between the observed and simulated landuse maps. Finally, four simulated future states were produced covering the BSC until the year 2050 for the following landuse classes: forest, grassland, cropland, built-up area, scrublands, crops/natural vegetation, and barren land.

Daily gridded precipitation and temperature datasets were created as well as scenarios of **climate change**. A two-step process was applied to obtain these gridded datasets. First we developed gridded precipitation and temperature (maximum and minimum values) data over the Black Sea catchment. These surfaces were created using the Integrated Nested Laplace Approximations (INLA) algorithm^17^ as implemented in the R-INLA package (more information on this package is provided in Code availability section). The surfaces were obtained by interpolation of the daily observations at meteorological stations collected in the Global Historical Climatology Network (GHCN) and European Climate Assessment & Dataset (ECA & D), covering the BSC over the period 1961–1990. The INLA interpolation validation was performed by means of cross-validation, both precipitation and temperature surfaces were interpolated using R-INLA with a spatial resolution of 0.25° for both longitude and latitude. In a second step we downscale the Regional Climate Models (RCMs) outputs from the PRUDENCE (Prediction of Regional scenarios and Uncertainties for Defining European Climate change risks and Effects). PRUDENCE was a European network program coordinate by the Danish Meteorological Institute (DMI), with the aim to create high-resolution regional simulations of present-day (1961–1990) climate and scenario simulations (2071–2100) for Europe. The scenario simulations were based on global climate models that simulate global climate response to the two regional IPCC's SRES GHG emission scenarios^18^. The two regional emission scenarios are the A2 and the B2 scenarios, and correspond in PRUDENCE to HS1 and HB1. From the available PRUDENCE outputs, we use the HIRHAM simulations (available online at http://prudence.dmi.dk/data/daily). Detailed information about how the regional simulation climate models were driven can be found on the PRUDENCE website (http://prudence.dmi.dk). The downscaling was made by using the Delta method. This method aims at perturbing observed time series with changes allowing increasing as a function of time. We derived downscaled climate projections by applying decile changes to perturb monthly rainfall and temperature probability distribution functions^19^. The validation of the Delta method was done by applying it first to the HIRHAM data set. The daily averages obtained for each scenario (HS1 and HB1) period were then subtracted from the corresponding daily averages of the present-day period (referred as HC1 in PRUDENCE). The differences are divided into deciles, and finally grouped into monthly deciles. This calculation is repeated for each climate scenario for the monthly precipitation and maximum and minimum values of temperature. The same methodology was later applied to the gridded surfaces to obtain the scenarios of climate change (2071–2100) for the BSC. Finally, we calculate the daily datasets 2025–2070 via linear interpolation between the last year from the control period (1990) and the first year of each scenario (2071).

We also integrated regional landuse and climatic change scenarios with **demographic data**. According to the enviroGRIDS storylines, the UN projection variants^20^ for population were analyzed, and a methodology was proposed for the downscaling of demographic data from national to regional level (NUTS2). Results included urban and total population trends over the period 2010–2050 for 214 regions. The data on population per region was collected for all available years from 18 different sources but mainly Eurostat and National statistical offices. Data were heterogeneous and occasionally incomplete. Therefore, a three-step process of harmonization was applied: (1) formatting and translating data from National statistical offices, (2) matching the statistical and geometric (boundaries) data together, and (3) interpolating linearly gaps for missing years. This process led to the completion of population data for the two base years (2001 and 2008) used in the landuse change analysis. Successively, missing values in time series were extrapolated linearly in order to cover the period 2000–2010. Considering that there is no uniform data on the proportion of urban population at the regional level, the urban population per region was calculated as the sum of cities with at least 10,000 inhabitants included in that region. To achieve this, a specific spatial data set containing population size of cities was generated. Except for EU 27 (ref. 21) and Russia, data for demographic projections at sub-national scale was not available. Therefore, the regional projections for total and urban population, for the period 2011–2050, were successively estimated on the assumption that future demographic developments can be derived from past population trends and hence, a continuation of observed demographic change is assumed. In the first step we analyzed how population is distributed in different regions of each country and used the trend to extrapolate until 2050. Once the trend was identified in a region over the period of interest, we transposed national United Nation Statistical Division population data for the three corresponding variants (high, medium and low fertility). Regarding the urban population, before applying the same approach, we first calculated the ratio between the urban and total national population. Subsequently, with the assumption that the urban growth rate is distributed uniformly between regions, the percentage of urban population was then calculated per region.

The Soil and Water Assessment Tool (SWAT2009; ref. 8) was used to develop the **agro-hydrological model** of the BSC. SWAT is a continuous-time and process-based hydrological model. We chose this program because it lends itself easily to climate and landuse change analyses and couples various processes to simulate natural and anthropogenic systems in watersheds including hydrology, climate, snow, nutrient, soil, sediment, crop growth, pesticide, surface depressions, and agricultural and water management. A water balance equation is solved to initiate water impoundment, which is a function of total inflow (e.g., runoff entering from the upstream subbasins, rainfall, groundwater contribution) and total outflow from the water bodies (e.g., evaporation, seepage into the subsurface). More details about the model are provided by in its theoretical manual^22^.

The BSC SWAT project was built with data that are described in details in a dedicated publication^23^ on observed^24,25^ and gridded^26,27^ precipitation and temperature, solar radiation^28^, soil characteristics^29^, landuse categories^30^, digital elevation model^31^, river networks^32^, estimated nutrients point sources, and agricultural management^33^. The spatial heterogeneity of the watershed was preserved by topographically dividing the basin into multiple sub-basins (12,982) using 90-m digital elevation model. These were further subdivided into hydrologic response units (HRU) based on the unique combinations of soil, landuse, and slope characteristics (89,202 HRU). These subdivisions enabled the model to reflect differences in evapotranspiration for various crops and soils. In each HRU and at each time step, the hydrologic and vegetation-growth processes were simulated based on the curve number rainfall-runoff partitioning and the heat unit phenological development method^22^. The agro-hydrological model was run at a daily time step where a full run took 42 h on a single Windows computer (64-bit, 2.7 GHz processor, 4 cores, and 8 GB of RAM). Calibration runs where, therefore, made in a network of 50 Linux computers^34^. The Hydrological model was calibrated against observations in river discharge, nitrate concentration, and crop yields across the catchment for the period of 1970–2000. Monthly river discharge data was obtained from Global Runoff Data Centre^35^, National Institute of Hydrology and Water Management (INHGA), Danube Delta National Institute for Research and Development (DDNI) in Romania, and Turkish Ministry of Forest and Water Affairs (MEF) (144 observation stations in total). Nitrate concentration in rivers was taken from International Commission for the Protection of the Danube River (ICPDR) (37 stations). Crop yield data was obtained from McGill University^33^ at 5 arc-minute resolution. The calibrated model was then tested with an independent set of data on river discharge, nitrate concentration, and crop yields for the period of 2001–2006.

We used the Sequential Uncertainty Fitting program SUFI-2 (refs 36,^37^) to calibrate and validate the model results. The program is linked to SWAT and provides the basis for parallel processing of multi-gauge calibration and large-scale parameterization schemes^24^. It also provides a platform for sensitivity and uncertainty analysis. In SUFI-2, parameter uncertainty is described by a multivariate uniform distribution in a parameter hypercube, while the output uncertainty is quantified by the 95% prediction uncertainty (95PPU) calculated at the 2.5 and 97.5% levels of the cumulative distribution of an output variable. Latin Hypercube is used to draw independent samples from the input parameter uncertainties, which lead to the calculation of the 95PPU for a give output variable. Two statistics quantify the goodness of fit and model output uncertainty. These are the *p-factor*, which is the percentage of measured data being bracketed by the95PPU, and *r-factor*, which is the average thickness of the 95PPU band^22,23^. The *p-factor* has the highest value of 1, while the lowest value for *r-factor* is zero. For flow, Abbaspour *et al.*^23^ suggest a practical value of 0.6–0.8 for the *p-factor* and a value around 1 for the *r-factor*. In this definition, (1-*p-factor*) can be thought of as model error, or measured data not accounted for by the model.

Having the calibrated BSC model in hand, water resources components such as **blue water** (fresh water availability), **green water flow** (evapotranspiration), and **green water storage** (water stored in soil) along with their associated uncertainties were calculated for the entire BSC. These concepts give an overall picture of water resources and bring the outputs of the BSC model closer to the needs of water resources researchers and policy makers. The suggested upper and lower bounds of the 95PPU reflect primarily the prediction uncertainty (including model structure, parameters, and inputs), as well as the temporal climate variation as a secondary effect. A comprehensive agro-hydrological database of the BSC comprising of all input data essential for building a detailed hydrological model as well as all the historic water resources modeling results was created and shared on the enviroGRIDS GeoNode platform to fill the existing gaps in water resources data in the region.

The vulnerability assessment aimed at assessing the **potential impact** of climate change on agricultural water resources, the adaptive capacity due to irrigation, and the resulting vulnerability of agricultural water resources in the BSC. The data used for the study is based on a agro-hydrological model discussed above^23^. Using SWAT^8^, two strong changing forces were applied under two scenario conditions: 1) the daily temperature was increased by 3 °C, and 2) the daily precipitation was decreased by 30%. The relevant model outputs were temperature, precipitation, irrigation, potential daily evaporation, and stream flow, all provided on a daily time step and for each of the 12,892 sub-catchments of the BSC. The related uncertainties concern the predictions of the agro-ecological model and have been discussed above. The output variables were used to compute auxiliary indicators such as temperature stress, water stress, and environmental water requirement, and were subsequently processed to produce indices for potential impact, adaptive capacity, and vulnerability. The **potential impact** was measured by the changes in natural plant growth days per year. A plant growth day was considered to be a day with neither water stress nor temperature stress. Temperature stress days were defined as days when the average daily air temperature was below 5 °C or above 35 °C^38^, whereas water stress days were defined as days when the actual daily evapotranspiration was less than half of the potential daily evapotranspiration^39,40^. Days when water stress would have occurred without the help of irrigation were not considered to be natural plant growth days. The **adaptive capacity** was measured by the number of potential irrigation days per year. A potential irrigation day was considered to be a day when the actual stream flow is above the environmental water requirement^41^. The latter was estimated by calculating 80% of the 10-year average stream flow for each respective day^42^. Finally, the **vulnerability** was measured by the change of the total number of plant growth days per year. Plant growth days were defined as the number of days with neither water stress nor temperature stress. Contrary to the number of natural plant growth days, the total number of plant growth days included days when plant growth was possible only with irrigation.

**Water scarcity indicators** aim at mapping and informing decision-makers and the public about the use of this limited renewable resource. In this section, we balanced modelled surface water availability in the BSC^23^ with needs and consumptive use of key water users, i.e., municipalities, power plants, manufacturing, irrigation^43^ and livestock breeding^44^. We took into account evaporation from major reservoirs and environmental flow requirements (EFR) to preserve critical ecosystem structures and functions. We evaluated EFR with the Variable Monthly Flow (VMF) method^45^ allowing for multiple disturbances to occur so that socioeconomic development can be supported. The needs and consumptive use of municipalities were calculated using urban and rural population densities, rate of connection to a running water supply system, and national domestic water withdrawal intensities. Water need for cooling power plants was calculated taking into account electricity production, fuel type, and cooling system of 3,065 power plants. Those for manufacturing production were acquired from national statistics offices, FAO^46^ and World Bank^47^ and downscaled according to urban population density. Net irrigation water requirements were obtained from Siebert and Döll^48^ and temporally allocated using the same crop coefficient method as in aus der Beek *et al.*^43^. A country specific irrigation efficiency factor was applied to obtain gross irrigation withdrawals. Withdrawals and consumptive use for livestock breeding were estimated using cattle, goat, sheep, pig and poultry densities, as well as dominant livestock production systems. In the absence of monthly data, withdrawals and consumption of all sectors except for irrigation were assumed to remain constant during the year, and to only vary from year to year. Evaporation from 325 large reservoirs was estimated using the Penman-Monteith equation assuming a daily evaporation 10% higher than reference evapotranspiration^49^. With this information, we modelled two complementary water scarcity indices. The first was about consumption (*WSI*_*c*_), and how the reduction of water flow could threaten downstream ecosystems and users following Hoekstra’s index^50^. The second (*WSI*_*w*_) was about demand, i.e., withdrawals, and how the available water could be insufficient, even independently from upstream consumptive use according to Smakhtin^51^. The following representative scales were used for *WSI*_*c*_<=0.2: No scarcity; 0.2–0.3: Low; 0.3–0.4: High; and >0.4: Very high scarcity; and for *WSI*_*w*_<0.1: No scarcity; 0.1–0.2: Low; 0.2–0.4: Moderate; 0.4–0.8: High; and >0.8: Very high scarcity.

**Sandy beaches** are accumulation zones of unconsolidated material in the shoreline, consisting of sediments transported by littoral drift. As natural and dynamic system, beaches provide many valuable ecosystem services such as coastal protection (sediment storage and transport, wave dissipation, buffer effect to extreme events, dynamic response to sea level rise), biodiversity preservation, water filtration and purification, and recreational landscape^52^. Being squeezed between terrestrial and marine environments, this ecosystem is particularly vulnerable to coastal erosion. On the marine side, sea is rising^53^ and extreme events (e.g., storm surge) could become change in frequency and intensity in some parts of the Black Sea^54^, in turn modifying long or short-term coastline readjustments that are impeded in an increasingly urbanized coastal environment^55^. Coastal erosion is also exacerbated by negative riverine sediment budget supply (sediment retention in dams, river’s water diversion, and decreased rainfall) coupled with sand mining and poor-design coastal protection engineering. This vulnerable ecosystem needs long-term field experiments and monitoring programs to describe its dynamics^52,56^ rather than one-shot descriptive database. However, we provided a first comprehensive digital inventory of Black Sea beaches at the basin scale obtained by photo-interpretation of freely available remote-sensed images on the web^11^. The resulting database contains 1,228 beaches (polygons) enriched with descriptive attributes (e.g., photo-based visual estimation of the sediment type, presence of coastal defences, urban development, and presence of dunes). Digitized polygons covered a total coastline length of 2,042 km with an area of 224 km^2^. Results showed that almost half of the Black Sea beaches are associated with artificial coastal protection schemes suggesting an already considerable beach erosion problem. Cross-shore beach retreat predictions due to sea level rise (SLR) were obtained by selecting the means of the lowest and highest projections estimated by an ensemble of six 1-D analytical and numerical models (Bruun^57^, Dean^58^, Edelman^59^, Leont’yev^60^, Xbeach^61^, SBEACH^62^: more details in Allenbach *et al.* 2016). All models were launched for the full range of plausible coastal environmental conditions encountered at the basin scale (i.e., using different combinations of wave conditions, sediment grain sizes and slope) for eleven scenarios of SLR (ranging from 0.1 to 2 m). Black Sea beach erosion risk was then evaluated by comparing the coastal retreat predictions for three SLR scenarios (0.5, 0.82 and 1 m) with the maximum widths of each beach. Selected scenarios corresponded to the approximate mean and high estimates of the mean SLR scenarios of the IPCC^63^ for the period 2081–2100. Finally, for a rapid assessment of the beach erosion risk, percentages of beach surfaces losses were calculated for the selected three SLR scenarios and added to the attribute table of the data set.

### Code availability

The climate gridded surfaces (1961–1990 and 2071–2100) were created using R Project for Statistical Computing, freely available under the GNU General Public License, and can be downloaded at https://www.r-project.org/. Multiple R-packages were used as necessary, while the main package used was the R-INLA (http://www.r-inla.org). Implementation details are available from the corresponding author upon request.

Demographic outputs have been generated using publicly available data. Algorithms are described in detail by de Bono *et al.*^5^

The Metronamica^6^ (ML v.4.2.2) software used for landuse change modelling is a proprietary package and is not freely distributed.

SWAT (SWAT2009 rev, 528) is a public domain package used for hydrological modeling that is actively supported by the USDA Agricultural Research Service at the Grassland, Soil and Water Research Laboratory in Temple, Texas, USA. (http://swat.tamu.edu/software/swat-executables). SWAT-CUP was used for calibration of SWAT and is freely available from (www.neprashtechnology.ca).

Water vulnerability and water stress indicators were developed with scripts in R and are available from the first author of the study upon request.

The models used to predict cross-shore beach retreat induced by long and short-term sea level rise scenarios were developed with MATLAB software by the team of Prof. A.F. Velegrakis from the Department of Marine Sciences from the University of the Aegean and are freely available through the RiVAMP methodology training material^64^.

The GeoNode open source platform version 2.0 has been used for sharing the enviroGRIDS data sets. This web-based application facilitates the visualization, download, sharing, and collaborative use of geospatial data through web services. It is freely available from the dedicated website (http://geonode.org/) under a GNU General Public License.

## Data Records

The entire data set is made freely available and accessible in various formats on the public domain. This includes a dedicated geoportal for data discovery, visualization and downloads, web services endpoints to ensure that data are accessible with interoperable standards (e.g., Open Geospatial Consortium (OGC) standards). Finally, a static copy of the data is made available at the data Dryad Repository (Data Citation 1).

The web-based interactive geoportal is accessible from the geoportal http://blacksea.grid.unep.ch. Users can select the Map tab to dynamically visualize and download datasets on: Landuse scenarios, Climate scenarios, Demographic scenarios, Hydrology, Water for agriculture vulnerability, Water scarcity, and Beach retreat.

Several vector formats are available such as: ESRI Shapefile, Geography Markup Language (GML) and Keyhole Markup Language (KML). Data in raster formats are available as Geotiff, ArcGrid, Gtopo30 and ImageMosaic. Metadata is available as: ATOM, Directory Interchange Format (DIF), Dublin Core, EBRIM, FGDC and ISO19115 formats.

In addition, several OGC-compliant web service endpoints are available to access graphical/cartographic representations of the data:

- Web Map Service (WMS): blacksea.grid.unep.ch/geoserver/wms?
- Web Map Tile Service (WMTS): blacksea.grid.unep.ch/geoserver/gwc/service/wmts?
- Vector data: Web Feature Service (WFS): blacksea.grid.unep.ch/geoserver/wfs?
- Raster data: Web Coverage Service (WCS): blacksea.grid.unep.ch/geoserver/wcs?
- Metadata: Catalog Service for the Web (CSW): blacksea.grid.unep.ch/catalogue/csw?

These interoperable web service endpoints enable user to seamlessly access and/or integrate these datasets in their desktop, web-based client, or own workflows. Moreover, the users are ensured to access the most up-to-date datasets. All the web service endpoints have also been registered into the GEOSS.

### Landuse change scenarios

**Figure 2:**
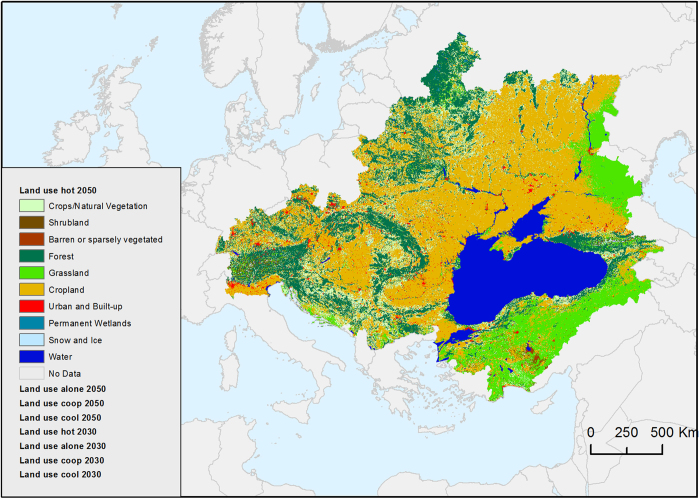
Landuse change for Scenario Black Sea Hot by 2050 *[blacksea.grid.unep.ch/maps/182]*.

The data consists of a set of nine raster maps representing the Landuse classification derived from MODIS in 2010 at a 500 m pixel resolution, and predicted with Metronamica until 2050 according to the HOT, ALONE, COOP and COOL scenarios (Fig. 2). Each map of LU is subdivided into 10 classes. Crops/natural vegetation, shrubland, barren or sparsely vegetated, forest, grassland, croplands and urban and built-up areas are changing over the years, whereas water, snow and ice, and permanent wetlands remain temporally unchanged (Landuse change scenarios, Data Citation 1).

### SWAT hydrological outputs

**Figure 3:**
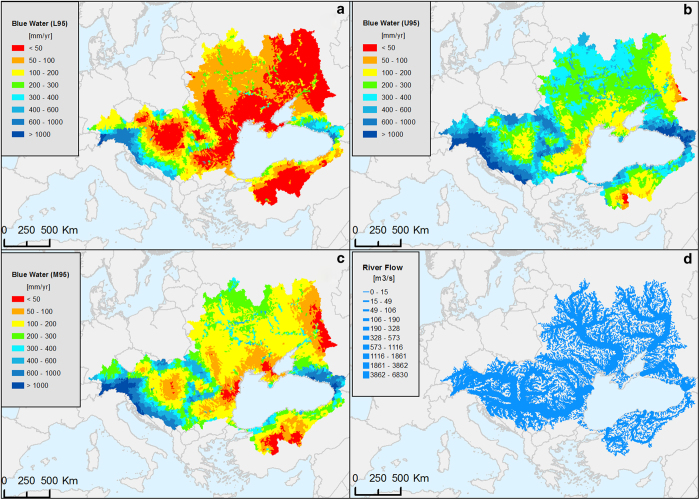
Outputs of the SWAT hydrological model of the Black Sea catchment. (**a**) Long-term annual average (1973–2006) of blue water expressed as lower (L95), (**b**) upper (U95) and (**c**) median (M95) of the 95% prediction uncertainty range. (**d**) River flow in m3/s. [blacksea.grid.unep.ch/maps/181].

The long-term annual average of blue water, monthly average river flow (Fig. 3) and nitrogen loads were simulated by the Black Sea SWAT model between years 1970 to 2006. These maps are based on 12,982 subcatchments and their corresponding river reaches. The available attributes are described in Table 1. (SWAT hydrological outputs, Data Citation 1).

### Climate

**Figure 4:**
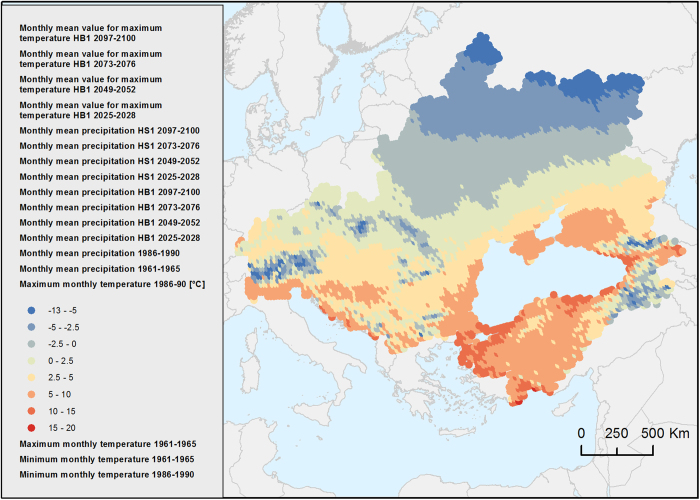
Map of Maximum monthly temperature *[blacksea.grid.unep.ch/maps/230]*.

This dataset consists of estimated values for monthly averages of maximum and minimum temperatures, as well as precipitation for the time period between 1961 and 1990, interpolated with the integrated nested Laplace approximations (Fig. 4). It also provides the estimated values of the monthly average for the HB1 and HS1 scenarios between 2071 and 2100, obtained through the Delta method. The dataset also includes the estimated values between 2025 and 2071 for both scenarios, obtained by means of a linear regression model (Climate, Data Citation 1).

### Demography

**Figure 5:**
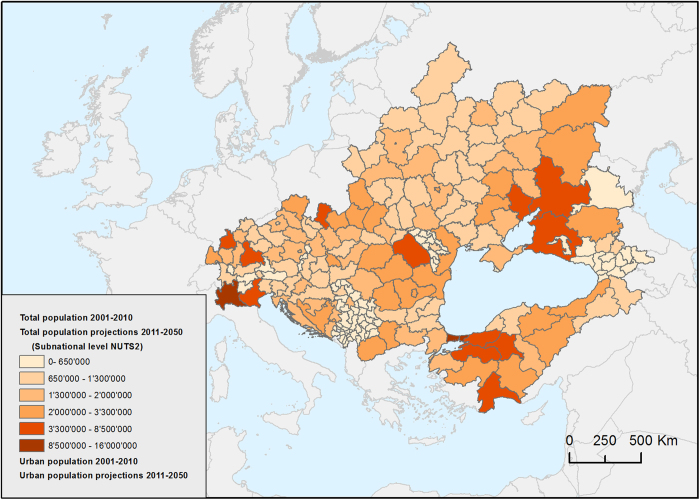
Total human population predicted for 2050 *[blacksea.grid.unep.ch/maps/228]*.

UN projection variants for population (Table 2), downscaled from national to regional level (NUTS2). Results include urban and total population trends over the period 2010–2050 for the 214 Black sea regions, consistent with BS HOT, BS ALONE, BS COOP and BS COOL scenarios (Fig. 5) (Demography, Data Citation 1).

### Water for agriculture vulnerability

**Figure 6:**
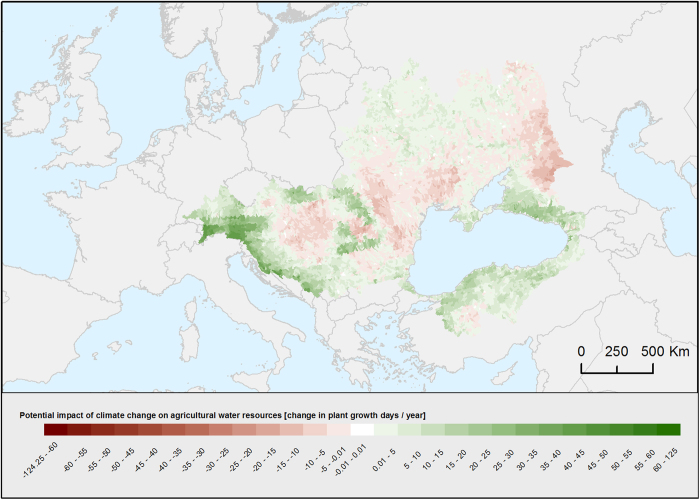
Potential impact of climate change on agriculture expressed as the change of annual plant growth days *[http://blacksea.grid.unep.ch/maps/231]*.

This map provides an overview of the potential impact of climate change on agriculture over 12,982 subcatchments. It is expressed as change in the annual plant growth days (Fig. 6). Green indicates an increase of plant growth days due to fewer days of water stress and/or fewer days of temperature stress (positive potential impact). Red indicates a decrease in plant growth days due to more days of water stress and/or temperature stress (negative potential impact). The potential impact is very heterogeneous across the BSC. The adaptive irrigation capacity and the total agriculture vulnerability is also made available in this data set (Water for agriculture vulnerability, Data Citation 1).

### Water scarcity

**Figure 7:**
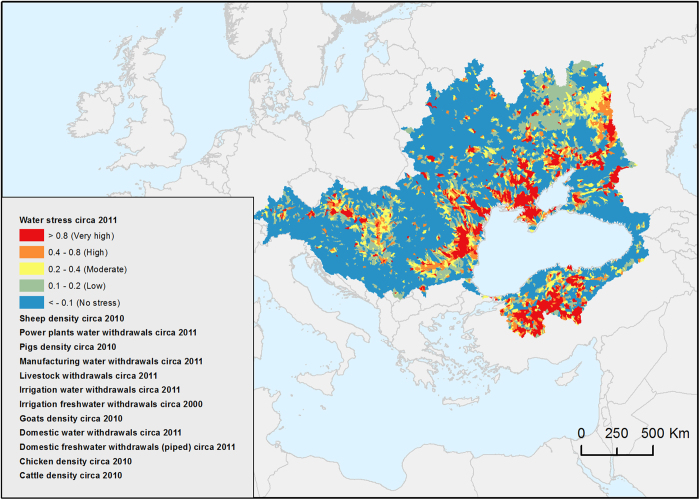
Water stress index by 2011 *[blacksea.grid.unep.ch/maps/232]*.

This aim of this data set is to represent water stress in the BSC circa 2011, as shown in Fig. 7. Water stress is calculated according to the Water Stress Index (WSI) corresponding to the quotient of total withdrawals by surface water availability, both in m^3^/yr. The scale of this index is as follows: <0.1=No stress; 0.1 to 0.2=Low; 0.2 to 0.4=Moderate; 0.4 to 0.8=High; >0.8=Very high stress. Several building blocks used to assess water stress are also made available as geospatial layers in this data set (Water scarcity, Data Citation 1).

### Beaches

**Figure 8:**
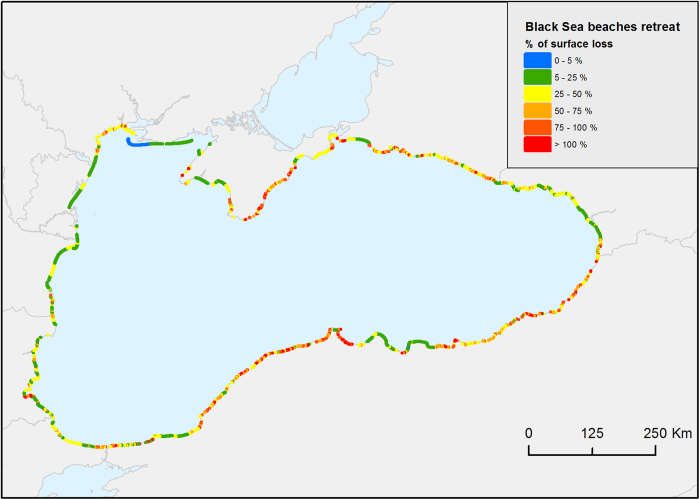
Predicted percentage of beach retreat of the Black Sea coasts *[blacksea.grid.unep.ch/maps/229]*.

Maximum beach retreat (in percentage of the maximum beach width) predicted by the model ensemble for the Black Sea beaches under a 0.5 m SLR (coastal retreat estimated to 21.4 m) is shown in Fig. 8. The attributes made available for each of the 1,228 digitized beaches are described in Table 3 (Beaches, Data Citation 1).

## Technical Validation

### Climate change

The technical validation of the gridded surfaces for the maximum and minimum temperature, and precipitation, was made by means of cross-validation with other interpolation models (such as thin plate splines), for a random selection of 1 day per month for 30 years, with 10 and 50% of the weather stations used for testing. The validation of the climate scenarios obtained with the DM was made by comparing the monthly average and standard deviation results obtained for both HS1(A2) and HB1(B2), with the gridded surfaces obtained by applying the delta method to the HC1.

### Landuse

During the calibration, the model parameters were repeatedly adjusted until the goodness of fit was optimized. Validation of the calibrated model comprised statistical procedures (Kappa statistic, Klocation, and Fuzzy Kappa), visual evaluation and stakeholders’ involvement in order to ensure its plausibility and accuracy.

### Population

Urban and rural downscaling of regional data were kept compatible with UN statistics for the period 2001 to 2010. Projections could not be validated but were in line with UN projections for the selected regions.

### Hydrology and SWAT outputs

The hydrological model was run at a daily time step (single run, 42 h). It was calibrated and validated for the period of 1970 to 2006 using observations on daily river discharges, river nitrate loads, and annual agricultural crop yields. Multi-objective, multivariable, multisite sensitivity, calibration, and uncertainty analysis were carried out using the Sequential Uncertainty Fitting program SUFI-2 (refs 36,^37^).

### Water for agriculture vulnerability

The main input for this analysis is the output of the SWAT hydrological model.

### Water scarcity

The main input for this analysis is the output of the SWAT hydrological model.

### Beaches

Digitalization based on photo interpretation, uncertainty analysis based on six different models of beach erosion. The uncertainty of the results can be appreciated by the difference between the max and min erosion percentages (SLR1max-SLR1min). Manual beach digitalization based on photo interpretation is subjective; accuracy was visually estimated at ±2 m. The lack of precise projection in the mosaicking images could induce shifts about 20 m between some images. Moreover, the completeness of the inventory is limited by the quality of available images (free of clouds and shadows).

## Usage Notes

A major caution should be taken concerning the resolution of the datasets. Indeed, the analysis was conducted using global/regional datasets, whose resolution are not relevant for local planning and should not be used for local decision-making. However, these datasets are intended to be used for national to regional assessments. They can be used also to compare countries or to help funding agencies to help them prioritizing environmental protection projects and investments.

In addition, some of these data sets result from models and depict plausible scenarios. Moreover, these scenarios are limited in spatial, temporal and thematic resolution due to present computing capacities.

Black Sea beaches data sets should be used being aware of their uncertainties induced by the methodology and data availability. Moreover, the inventory does not reflect the dynamic environment of this ecosystem that is submitted to strong natural fluctuations. The available remote sensing images are not synoptic at the basin scale. They have been collected in different years and seasons (within the period October 2000—January 2011) and during different wave run-up conditions.

All layers are publicly and freely available using widely used interoperable standards and are compliant with the GEOSS Data Sharing Principles:

1. There will be full and open exchange of data, metadata, and products shared within GEOSS, recognizing relevant international instruments and national policies and legislation.
2. All shared data, metadata, and products will be made available with minimum time delay and at minimum cost.
3. All shared data, metadata, and products being free of charge or no more than cost of reproduction will be encouraged for research and education.

These principles are central to the GEOSS vision ‘to realize a future wherein decisions and actions for the benefit of humankind are informed via coordinated, comprehensive and sustained Earth observations and information’.

By making these datasets available following GEOSS data sharing principles and using standardized web services, they can assist decision makers for environmental policies planning (e.g., INSPIRE Directive), environmental resource management (e.g., Water Framework Directive), support assessment processes of regional to local environmental agencies (like the International Commission for the Protection of the Danube River (ICPDR) or the Black Sea Commission (BSC)), and finally contribute to other web-based environmental platforms such as the DRDSI lead by JRC (http://drdsi.jrc.ec.europa.eu) or SCOPED-W^65^.

## Additional Information

**How to cite this article:** Lehmann, A. *et al.* A web platform for landuse, climate, demography, hydrology and beach erosion in the Black Sea catchment. *Sci. Data* 4:170087 doi: 10.1038/sdata.2017.87 (2017).

**Publisher’s note:** Springer Nature remains neutral with regard to jurisdictional claims in published maps and institutional affiliations.

## Supplementary Material



## Figures and Tables

**Table 1:** Attributes definition for rivers and subcatchments of the Black Sea catchment.

**Subbasin**	**Sub basin ID**
AreaC	Area drained by reach (km2)
Len2	length of river segment (m)
Slo2	slope of river segment (%)
Wid2	width of river segment (m)
Dep2	depth of river segment (m)
MinEl	minimum elevation of river segment (m)
MaxEl	minimum elevation of river segment (m)
Annual	Yearly average flow (m3/s)
Jan to Dec	Monthly average flow (m3/s)
Annual	Yearly nitrogen load (kg)
Jan to Dec	Monthly nitrogen load (kg)

**Table 2:** Attribute definition for population of the Black Sea sub-regional units.

**Nuts2_Id**	**Nuts id**
Name	Name of region
Y2011	Yearly projected population for 2011
Y2025	Yearly projected population for 2025
Y2050	Yearly projected population for 2050

**Table 3:** Attribute definition for beaches of the Black Sea coast.

**NAME**	**Code name of the beach, 3 first letters are the country followed by a clockwise dialing**
ORIENT	Calculated orientation of the beach in degree
LENGTH	Calculated shoreline length of the beach in meters
MAX	Calculated maximum width of the beach in meters
MEAN	Calculated mean width of the beach in meters
AREA	Calculated area in square meters
DATE	Date of the satellite image used
SLR1min	minimum % of the beach surface loss by a coastal retreat of 4.1 meters induce by a SLR of 0.5 m
SLR1max	maximum % of the beach surface loss by a coastal retreat of 21.4 meters induce by a SLR of 0.5 m
SLR2min	minimum % of the beach surface loss by a coastal retreat of 6.9 meters induce by a SLR of 0.82 m
SLR2max	maximum % of the beach surface loss by a coastal retreat of 31.6 meters induce by a SLR of 0.82 m
SLR3min	minimum % of the beach surface loss by a coastal retreat of 8.5 meters induce by a SLR of 1 m
SLR3max	maximum % of the beach surface loss by a coastal retreat of 37.3 meters induce by a SLR of 1 m
